# Spread of volunteer and feral maize plants in Central Europe: recent data from Austria

**DOI:** 10.1186/s12302-016-0098-1

**Published:** 2016-12-30

**Authors:** Kathrin Pascher

**Affiliations:** Division of Conservation Biology, Vegetation- and Landscape Ecology, Department of Botany and Biodiversity Research, University of Vienna, Rennweg 14, 1030 Vienna, Austria

**Keywords:** Maize, Corn, Genetically modified (GM), Feral plants, Volunteers, Central Europe, Transgene spread, Field observations, Ecological risk assessment (ERA), EU legislation

## Abstract

The occurrence of volunteer maize plants in subsequent crops as well as of feral maize plants in non-agricultural areas is an essential issue in risk assessments of genetically modified (GM) maize, with regard to possible contamination of natural habitats with GM material and as contribution to the total adventitious GM content of the non-GM final product. The appearance of feral maize plants has been confirmed for non-agricultural habitats in European areas with Mediterranean climate such as Spain. However, the existence of maize volunteers and feral maize outside cultivation under Central European continental climatic conditions is considered to be extremely unlikely in those winter-cold areas. Here, field observations during 5 years (2007, 2008, 2010, 2011 and 2015) in Austria are presented that confirm the occurrence of volunteer and feral maize under Central European climatic conditions. Most of these plants produced fertile inflorescences with viable pollen and fully developed cobs. Maize kernels may reach the soil by disintegration of cobs due to disease, using crushed maize cobs for game-feeding, left overs in manure dispersed during fertilisation or from transporting and handling of crushed cobs. The evidence of volunteer and feral maize in four Federal States in Austria (Burgenland, Lower Austria, Upper Austria, Styria) emphasises the necessity to consider these hitherto under-emphasised factors in an ecological risk assessment (ERA) of GM maize as a possible source for transgenes in non-agricultural habitats, because these plants could act as bridge for the spread of GM material into semi-natural habitats. In accordance with the European Food Safety Authority (EFSA), which states that in principle maize has the potential to survive as a volunteer or feral plant also in regions with cold winters, the investigation of the frequency of their occurrence under Central European conditions should be part of future monitoring programmes in order to assess their potential for permitting transgene spread.

## Background

Maize (*Zea mays* subsp. *mays*) is an annual monoecious crop frequently grown in many countries. In 2014, a total area of 184 Mio hectares was cultivated worldwide (http://faostat3.fao.org/download/Q/QC/E, accessed 24th of July 2016). Currently, around 30% of maize is genetically modified (GM) [1]. In 2014, 143,016 hectares of biotech Bt maize Mon810 have been cultivated in the EU, mainly in Spain. Transgenic maize for commercial production confers either insect resistance or herbicide tolerance or a combination thereof. This crop is mainly used for food and livestock feed, but also for renewable resources. Maize, domesticated by native Indians of Mexico and northern Central America already about 5500 years ago [2], has been introduced to Europe in 1525 owing to the discovery of America by Columbus. Since then, a large number of local varieties have been developed all over Europe. This crop has also been subject to trait improvement via genetic modification since several decades. In case of cultivation of GM maize, the main factors that determine adventitious presence of a genetically modified organism (GMO) in non-GM material are unintended seed impurity, seed planting equipment and practices, cross-pollination between GM and non-GM crops, the presence of GM volunteers, and product mixing during harvest, transport and/or storage processes [3]. Moreover, due to the current focussing in breeding, improvement and use of only a few crop varieties, the diversity of maize landraces could be threatened in future. Cross-pollination is possible in areas with hybridisation partners such as Mexico. However, teosinte—the closest relative of maize—has recently been detected also in Spain where it behaves like an invasive weed of agricultural land (http://www.agpme.es/index.php?option=com_content&view=article&id=181:el-teosinte&catid=44:articulos&Itemid=68, accessed 30th of July 2016). Even though maize is a mainly wind-pollinated crop [4], it has also been observed to function as pollen source for honey bees [5]. So, non-target organisms that collect pollen of maize plants are exposed directly to GM pollen. Additionally, volunteer and feral maize plants contribute to a prolonged GM pollen exposure. Hence, the relevant environmental aspects of volunteer and feral maize include uncontrolled dispersal of GM plants into the environment, prolonged exposure of non-target organisms to GM pollen, increased use of herbicide to remove volunteer and feral maize and an adopted insect resistance management that is mandatory for Bt crops. In the USA, volunteer maize growing in soybean fields above the soybean canopy is known as a highly competitive weed and requires specific herbicide application [6].

It is controversially debated among European scientists, stakeholders and policy makers, whether maize volunteers in subsequent crops may pose a problem also in colder climatic zones of Europe. Moreover, it has been questioned, if maize has the ability at all to become feral outside cultivation in areas with cold winter temperatures and how the likelihood of becoming feral has to be rated under Central European continental climate conditions compared to those in Mexico. Some scientists assume that maize as a highly domesticated crop has very little invasion potential and poses a negligible ecological risk [7]. Maize seeds and seedlings are assumed to survive the winter only in southern European countries, such as Spain, where maize kernels that remain on the soil after harvest can germinate and develop into flowering individuals, which can locally cross-pollinate neighbouring maize plants [8]. However, by a combination of weak growth, asynchronous flowering with the maize crop, low resistance to frost, low competitiveness, absence of a dormancy phase, susceptibility to diseases, herbivory and cold climate conditions survival of the plants is estimated to be unlikely, rendering the risk for outcrossing and establishment of populations limited [3, 9, 10]. So far, there have been no records for survival of volunteer and feral maize plants in the Netherlands [11]. Occasional records for maize growing outside agronomic conditions on the British Island have been made, but are rare [12, 13]. Irish maize varieties, while cold adapted, were observed to be still frost intolerant [14]. However, single plants were registered in two Irish port locations, Limerick and Dublin [15]. In contrast, in an American study, several volunteer maize kernels were found to be winter-hard in northern latitudes and germinated the following spring [16]. Even in Germany, GM volunteer maize plants—containing the Nos-terminator and the CaMV35S-promotor—were recorded for the first time on a field of Monsanto in Nordrhein-Westfalen in 2007 (http://www.proplanta.de/Agrar-Nachrichten/Wissenschaft/GVO-Mais-ueberwintert-erstmals-in-Deutschland_article1185528877.html; http://www.zeitpunkt.ch/news/artikel-einzelansicht/artikel/durchwuchs-gentech-mais-ueberwintert-erstmals-in-deutschland.html; http://www.haerlin.org/Mais_Durchwuchs.pdf, accessed 24th of July 2016). The GM maize had been seeded in 2006 and several seeds obviously survived the mild winter temperatures in 2006/2007. It is stated that climate change could be a driving force for overwintering of maize seeds in future.

The term “to become feral” in the context of a crop refers to the crop’s occurrence outside cultivation. The invasiveness potential of a crop is the likelihood that it will persist and spread in non-agricultural habitats [7]. Ecological harm in connection with a GMO includes that the transgenic crop produces seeds, which then disperse to non-agricultural habitats, that the crop establishes in the non-agricultural habitat and forms a self-sustaining population. If feral plants spread and thereby influence the abundance of native species, they will cause ecological harm [17–19]. It is often argued—because no visible ecological harm has been identified during the long history of cultivation of the conventional crop-type—that there would be no negative effect originating from the GM crop. It is assumed that the chain of the above listed events from cultivation to ecological harm is obviously broken at one or more links [7]. An Irish Study [14] says:

“E*vidence for this can be seen in the lack of anecdotal evidence supporting the existence of feral maize populations. It is safe to conclude therefore that under current climatic conditions and in the absence of selection pressure there is no likelihood of GMHT maize persisting over adjacent flora and hence there would be no detrimental impact on the Irish landscape should GMHT maize seed be lost pre*-*sowing*”.

Also the Organisation for Economic Cooperation and Development, OECD, is very sceptical towards a potential invasiveness of the crop maize [20]:

“*Volunteers are common in many agronomic systems, but they are easily controlled; however, maize is incapable of sustained reproduction outside of domestic cultivation*”.

The Netherlands Commission on Genetic Modification, COGEM [11] states:

“*During its long domestication process, maize has lost its ability to survive in the wild. In the Netherlands, the appearance of maize volunteers is rare and establishment of volunteers in the wild has never been reported. There are no reasons to assume that the introduced trait will increase the potential of maize to establish feral populations”.*

Contrastingly, other scientists consider volunteerism and ferality of maize as at least in principle possible [3, 21, 22]. Even the European Food Safety Authority (EFSA) gives the following statement concerning the occurrence of volunteer maize:

“*Maize is highly domesticated and generally unable to survive in the environment without management intervention. Maize plants are not winter hardy in many regions of Europe; furthermore, they have lost their ability to release seeds from the cob and they do not occur outside cultivated land or disturbed habitats in agricultural landscapes of Europe, despite cultivation for many years. In cultivation, maize volunteers may arise under some environmental conditions (mild winters). Observations made on cobs, cob fragments or isolated grains shed in the field during harvesting, indicate that grains may survive and overwinter in some regions, resulting in volunteers in subsequent crops. The occurrence of maize volunteers has been reported in Spain and other European regions*” [8, 23].

The present article will contribute to the debate whether maize is able to become feral and to exist as a volunteer plant in Central Europe, exemplarily shown with Austrian data. In Austria, a temperate Central European transition climate is predominant with a continental climate in the east of the country and influences of the oceanic climate in the west. Large climate differences exist between the moderate climate in the Alpine north and the Mediterranean influences in the Alpine south. Austria is rich in diversity of landscapes and of animal and plant species [24, 25]. A release of GM crops has been performed neither for field experiments nor for cultivation in this EU member state. Several proofs (photographs taken during fieldwork) of the occurrence of volunteer and feral maize plants in Austria will be presented here.

## Methods

All records reported here were made by accident during fieldwork for three studies in Austria. The study BINATS [25] covered altogether 100 test areas, each 625 × 625 m in size; 50 test areas were located in maize cultivation regions (Lower and Upper Austria, Burgenland, Styria, Carinthia) and 50 in oilseed rape cultivation regions (Lower and Upper Austria, Burgenland). For selection of test areas, a stratified random sampling procedure for monitoring biodiversity in the Austrian agrarian regions was applied, including criteria such as diversity of soil types, forest cover in close proximity to the test area, grassland cover, average annual temperature or average annual precipitation. In the study FEAR [26], 50 potato fields and 50 maize fields were selected randomly, but representative for the extent of cultivation and diversity of soil types in the Austrian potato and maize cultivation regions. The maize fields investigated for FEAR were located in the 50 BINATS maize test areas. The maize growing region of Lower and Upper Austria were sampled more intensively than those of the other Federal States (Burgenland 6, Styria 4, Carinthia 2). Similarly, most sampling sites from the potato growing area were from Lower and Upper Austria, fewer fields were investigated elsewhere (Styria 3, Tyrol 3, Burgenland 1, Salzburg 1). For the third study, dealing with imported oilseed rape [27], 60 investigation sites were selected all over Austria including presumable hotspots for seed spillage such as switchyards (2), border railway stations (6), main ports (3), OSR importing oil mills (3) and an OSR processing facility (1) as well as randomly selected road sectors (2 kilometres; 22), railway stations (20) and small ports (3). Most of the sites were located in those Federal States where oilseed rape is mainly grown (Upper Austria 25, Lower Austria 11, Burgenland 2), fewer in the other Federal States (Salzburg 7, Styria 5, Tyrol 4, Vorarlberg 4, Carinthia 1, Vienna 1). As none of the sampling sites had been selected on expectations for the occurrence of volunteer and feral maize, the data presented here provide anecdotal evidence for the existence of volunteer and feral maize under Central European conditions at several locations in Austria and in several years, but they do not allow any assessment on regional distribution and abundance to be made.

## Results and discussion

### Occurrence of volunteer and feral maize plants in Austria

**Fig. 1:**
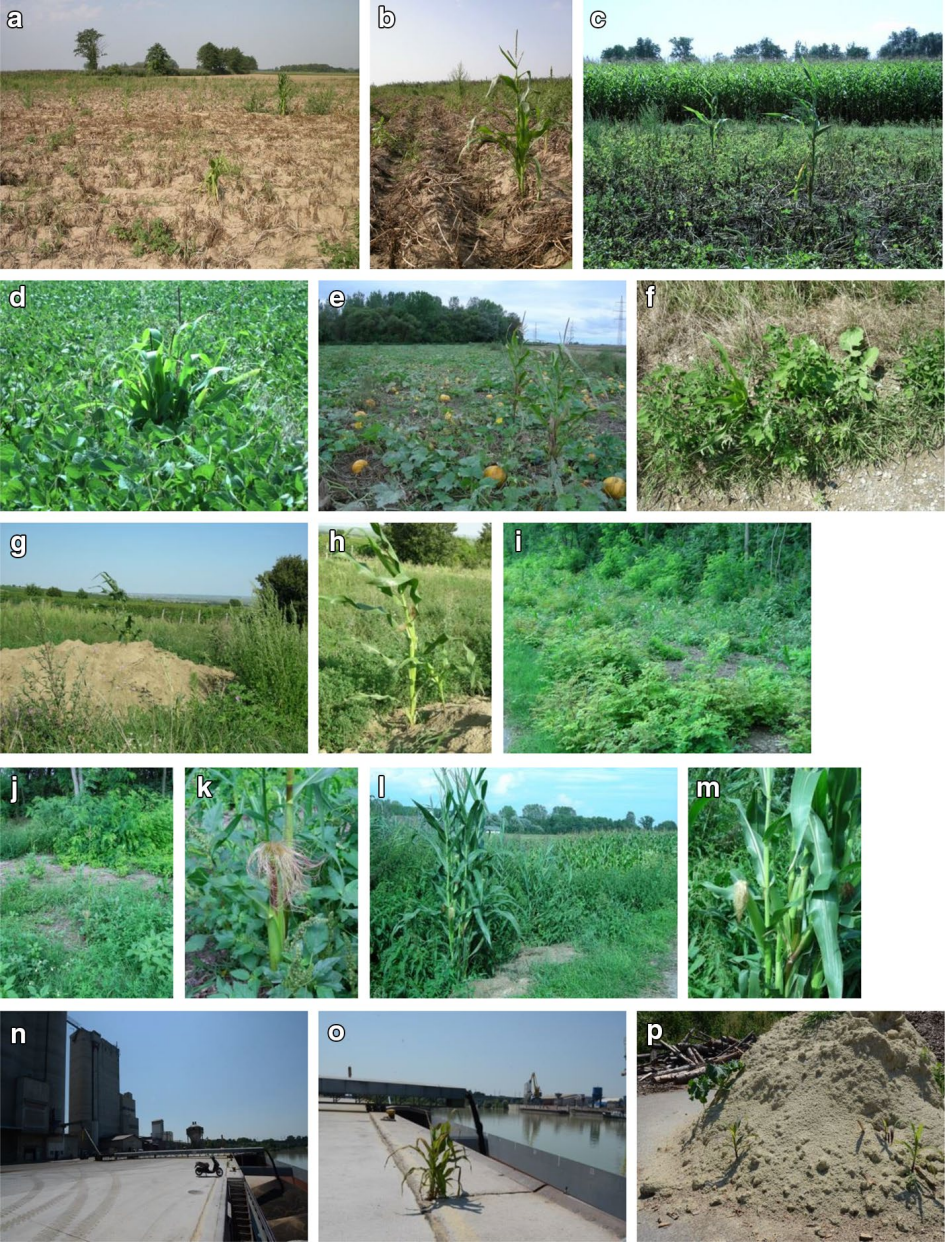
Observations of volunteer (**a**–**e**) and feral (**f**–**p**) maize in Austria. Volunteer maize: **a**–**c** potato fields near Bad Radkersburg in Styria (7th August 2011); **d** soybean field in Landegg close to Hornstein in Lower Austria (11th August 2011); **e** pumpkin field in Hausleiten in Lower Austria (9th September 2011). Feral maize: **f** Hornstein, Burgenland (18th August 2007); **g** and **h** Purbach am Neusiedlersee, Burgenland (12th August 2008); **i**–**k** at the edge of the “Zitzmannsdorfer Wiesen”, Neusiedlersee, Burgenland (19th August 2010); **l** and **m** Nestelbach, Styria; **n** and **o** loading area in the port of Enns, Upper Austria (12th August 2015); **p** pile of sand located in the port of Enns, Upper Austria (12th August 2015), moreover feral oilseed rape plants could be observed on the pile

**Fig. 2:**
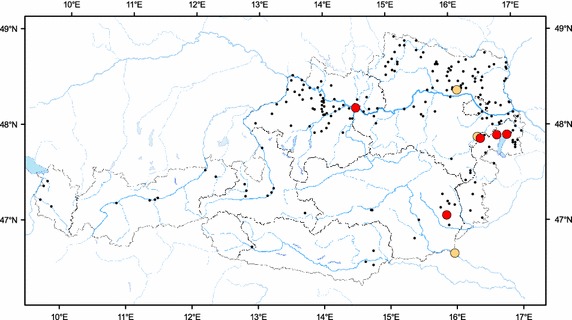
Austrian map with spots of discovery of volunteer and feral plants. Volunteer maize (marked with *light*-*orange spots*): Radkersburg in Styria, Landegg bei Hornstein in Lower Austria, Hausleiten in Lower Austria. Feral maize (marked with *red spots*): Hornstein in Burgenland, Purbach in Burgenland, “Zitzmannsdorfer Wiesen” in Burgenland, Nestelbach in Styria, port of Enns in Upper Austria. The locations of altogether 210 test areas/sampling sites of three Austrian studies (BINATS, FEAR, study dealing with imported oilseed rape) are indicated in the map with *small black spots*

Volunteer maize plants were observed in two potato fields in Styria as well as in a soybean and a pumpkin field in Lower Austria in summer 2011 (Figs. 1a–e, 2) during field sampling in the course of the project FEAR [26]. Several of the volunteer plant individuals found—seven and ten, respectively—flowered and had already produced vital cobs.

Feral maize plants were observed in three Austrian Federal States (Burgenland, Styria and Upper Austria; Fig. 2) in August in the years 2007, 2008, 2010 and 2015 during fieldwork for three studies [25–28]. Most of the feral plants—one individual in Hornstein (Fig. 1f; Burgenland); two individuals in Purbach am Neusiedlersee (Fig. 1g, h; Burgenland); around 30 individuals at the “Zitzmannsdorfer Wiese” (Fig. 1i–k; Burgenland); six individuals in Nestelbach (Fig. 1l, m; Styria); three individuals at the unloading area of the port of Enns (Fig. 1n, o, Upper Austria) and three individuals on a sand pile at the port of Enns (Fig. 1p)—were fertile and had already produced cobs. Particular emphasis has to be put on the observation of the highest number of observed feral maize plants on the edge of the “Zitzmannsdorfer Wiesen” as this area is part of the National Park Neusiedler See—Seewinkel. Like the locations Hornstein and Purbach, the National Park belongs to the Pannonian climate region. As can be seen in Fig. 1i–k, the plants have grown on a rather open site together with the black locust (*Robinia pseudoacacia*) which is known as an especially aggressive invasive species [29]. Most of the feral maize plant individuals were observed in the warmer Pannonian region. Most records were from Burgenland and Styria, although density of sampling sites was much higher in Lower and Upper Austria, i.e. record density is not correlated with sampling density. Feral plants have further been found at the port of Enns where loading of maize seeds is regularly performed (Fig. 1n–p). Single-maize kernels are handled there and loaded on ships for further transportation. After loading, the storage areas of maize kernels are cleaned with brushes. If single-maize kernels remain in that area in spite of cleaning, they have the potential to germinate and develop a fertile plant.

Commercial maize has lost its ability to release single kernels from the cob. Hence, single-maize kernels are rare in fields and spillage of them probably mainly traces back to seeding and harvest activities of farmers. Additional factors such as storm damage, poor stalk quality, insect damage and plant diseases can lead to kernel and ear losses which might result in volunteer maize in the following year [16]. Maize kernels are used as feed stuff for pigs, poultry or cattle fattening. Left overs reach the manure and in this way are dispersed into the environment during fertilisation. Feeding of game (e.g. wild boars) by hunters or fowl kept in an animal husbandry could be another source for the entry of single-maize kernels into semi-natural and natural habitats. For better feeding, the cobs are threshed into single components. This was probably also the case at the sampling site “Zitzmannsdorfer Wiesen”. No maize field was present in the surroundings of this ruderal habitat in the year of observation. Hence, it is likely that the feral plants originated from maize kernels used for game-feeding. Hunters sometimes cultivate fields for game browsing and protection against enemies. Single-maize plants are also part of this animal feed stuff. In a study in Korea [21], imported maize kernels were found to be usually processed and mixed with other components in the animal feed manufacturing plants, and finally consumed in the livestock barns.

### Records of volunteer and feral maize plants in other countries

In a study conducted in Spain [3], the number of maize volunteers differed strongly between twelve tested fields ranging from low (30 plants/ha) to extremely high numbers (>8000 plants/ha), thus accounting for nearly 10% of the total plants in the field. This variability in numbers was caused by many factors such as climate conditions in winter and early spring and applied agricultural practices (tillage, etc.). For instance, remnant maize kernels can suffer loss of vigour due to unfavourable weather in winter, may be at different depths in the ground and frequently lack optimal conditions for germination. It was observed that dry conditions during autumn favoured overwintering of non-germinated seeds in the fields.

In Spain, most of the volunteers generally did not produce any cob. If they did, the cobs were small and poorly pollinated. In contrast, most of the volunteers as well as several of the feral plants in the Austrian observations developed normal inflorescences and cobs with regularly developed kernels. They had normal vigour. Moreover, the plants did not show infestation but had a healthy appearance. The occurrence of these plants during several field study years does not mandatorily correspond to exceptional years with milder winter temperatures in Austria (www.zamg.ac.at, accessed 24th of July 2016).

Maize is commonly handled and transported as kernels threshed from the cob. Feral maize plants are able to develop from spillage events in course of seed loading in ports (Fig. 1n–p). The occurrence of feral GM maize as a result of kernel spillage during import, transport, storage, handling and processing activities was also confirmed for Korea, a country where no GM crop has recently been cultivated [21, 30, 31]. In the study of Kim et al. one GM maize plant was identified in a small vegetable garden in 2005 [30]. As a result of seed spillage, several GM maize plants were found along the roadside in the following year at a grain receiving port and around cultivated fields [31].

Moreover, several spilled maize kernels were observed around open storage areas of two ports and along truck transportation routes near feed manufacturing plants [21]. The monitoring sites focussed on retriever routes of imported maize from grain receiving ports to feed manufacturing plants and finally to livestock barns. While 120 kernels were found at or around the Incheon port—but no feral maize plants grew there—, 18 established feral maize individuals were registered at the Gunsan port. Fifteen of those were identified to have originated from GM varieties. Moreover, additional eight GM maize plants grew around four feed manufacturing plants and in two livestock barns. These findings prove that conventional as well as GM maize kernels are spilled during transportation and handling, and that both have the potential to develop fertile plants.

Maize has been cultivated in Europe for hundreds of years, but there is no indication so far that it has become an established weed even in countries with warmer climates despite genetic diversity of types and improvements. Although herbicide tolerance in maize, a selective advantage in habitats with herbicide application, is already known to cause problems [16, 32–34], GM maize is still considered of limited concern in the context of invasive weeds, at least outside agricultural systems. However, this might change, if maize became better adapted to cold climatic conditions. Introduced artificial traits such as cold or frost tolerance could trigger a different behaviour of GM maize compared to its conventional counterparts. Several risk hypotheses for a transgene spread into non-agricultural habitats via feral and volunteer maize plants are already discussed [19]. Although experiments did not yet provide evidence for an increased risk of transgene spread via feral and volunteer maize, such rare events may still be evolutionarily significant and their frequency might have actually changed with climate change. Concerning the appearance of feral plants in Central Europe and the existence of hybridisation partners such as teosinte in Spain, the ecological risk of GM maize has obviously changed maybe due to warmer winters. Hence, a new risk assessment is urgently needed.

In contrast to oilseed rape—a crop originating from Central Europe—with very frequent occurrence of feral plants and volunteers in Austria [26, 35], maize also produces feral plants and volunteers in subsequent crops but with lower frequency. Because maize exhibits about 95% cross-fertilisation [21], it might cause a high outcrossing rate. Hence, it is realistic that GM contaminations descending from volunteer as well as from feral GM maize in organic and conventional maize fields have to be expected in a region where GM maize is cultivated or imported and will contribute to the total adventitious GM content in final products.

## Conclusions

As a next clarifying step, it has to be investigated in detail if maize is also able to form self-sustaining populations outside cultivation and persist for subsequent years as a population. Although less probable in comparison to the crop oilseed rape, this essential aspect for transgene spread of GM maize has to be considered in ERA in future, especially in warmer areas such as the Pannonian region as shown here from observations in Austria. Additionally, detailed systematic and quantitative studies are needed to be able to verify if the maize plants persist over longer time periods or are transient. It is recommended that systematisation of research all over Europe should be performed in order to quantify the occurrence of feral and volunteer maize in regions with different winter temperatures.

## Abbreviations

COGEM: Commission on Genetic Modification
EC: European Commission
EFSA: European Food Safety Authority
ERA: ecological risk assessment
EU: European Union
GMO: genetically modified organism
GM: genetically modified
OECD: Organisation for Economic Cooperation and Development
